# Non Thermal Irreversible Electroporation: Novel Technology for Vascular Smooth Muscle Cells Ablation

**DOI:** 10.1371/journal.pone.0004757

**Published:** 2009-03-09

**Authors:** Elad Maor, Antoni Ivorra, Boris Rubinsky

**Affiliations:** 1 Biophysics Graduate Group, University of California, Berkeley California, United States of America; 2 Department of Mechanical Engineering, University of California, Berkeley, California, United States of America; 3 School of Computer Science and Engineering, Hebrew University of Jerusalem, Jerusalem, Israel; Leiden University Medical Center, Netherlands

## Abstract

**Background:**

Non thermal Irreversible electroporation (NTIRE) is a new tissue ablation method that induces selective damage only to the cell membrane while sparing all other tissue components. Our group has recently showed that NTIRE attenuated neointimal formation in rodent model. The goal of this study was to determine optimal values of NTIRE for vascular smooth muscle cell (VSMC) ablation.

**Methods and Results:**

33 Sprague-Dawley rats were used to compare NTIRE protocols. Each animal had NTIRE applied to its left common carotid artery using a custom-made electrodes. The right carotid artery was used as control. Electric pulses of 100 microseconds were used. Eight IRE protocols were compared: 1–4) 10 pulses at a frequency of 10 Hz with electric fields of 3500, 1750, 875 and 437.5 V/cm and 5–8) 45 and 90 pulses at a frequency of 1 Hz with electric fields of 1750 and 875 V/cm. Animals were euthanized after one week. Histological analysis included VSMC counting and morphometry of 152 sections. Selective slides were stained with elastic Van Gieson and Masson trichrome to evaluate extra-cellular structures. The most efficient protocols were 10 pulses of 3500 V/cm at a frequency of 10 Hz and 90 pulses of 1750 V/cm at a frequency of 1 Hz, with ablation efficiency of 89±16% and 94±9% respectively. Extra-cellular structures were not damaged and the endothelial layer recovered completely.

**Conclusions:**

NTIRE is a promising, efficient and simple novel technology for VMSC ablation. It enables ablation within seconds without causing damage to extra-cellular structures, thus preserving the arterial scaffold and enabling endothelial regeneration. This study provides scientific information for future anti-restenosis experiments utilizing NTIRE.

## Introduction

Vascular smooth muscle cells (VSMC) are the cellular component of the medial layer of adult arteries and play a prominent role in arterial physiology and pathology. [1], [2] Together with arterial remodeling, VSMC proliferation plays a major role in luminal loss following angioplasty.[2], [3] VSMC proliferation is even more important in the clinical scenario of in-stent restenosis, where remodeling is prevented by the firm scaffolding of the stent.[4]

To date, different methods to stop the proliferation of VSMC have been suggested. These methods include cryoplasty, brachytherapy, photodynamic therapy, drug-eluting stents and genetic manipulations using gene-therapy. Drug-eluting stents have shown the greatest clinical benefit[5], although there are recent concerns about the safety of this modality due to reports regarding the augmentation of late in-stent thrombosis and possible increased mortality.[6]

Irreversible Electroporation (IRE) is a phenomenon in which high electrical fields are delivered across cells in short, micro to millisecond pulses. These pulses create irreversible defects (pores) in the cell membrane lipid bilayer, causing cell death through loss of cell homeostasis.[7] Irreversible electroporation is associated with high electrical fields and is often reported in conjunction with tissue thermal damage caused by electrical Joule heating.[8]–[10] Joule heating is the dissipation of energy when an electrical current flows through a medium with resistance to current.

Both irreversible electroporation and thermal damage are a function of many parameters, such as electric field intensity and duration and thermal diffusivity. Our group hypothesized that it is possible to find a range of parameters that will cause only irreversible electroporation effects without thermal damage in a volume of tissue.[11], [12] Non Thermal Irreversible Electroporation (NTIRE) operates in such a mode, selectively affecting only the cell membrane lipid bilayer and sparing all the other molecules in the volume of the treated tissue.[12], [11] An acute experimental study supported by mathematical analysis demonstrated the concept in vivo.[13] The first fundamental long term study in a pig liver demonstrated the hypothesis as well as the advantages of NTIRE.[14] It was shown that NTIRE protocols can be designed for treatment of a volume of tissue and that while all the cells in the treated volume are uniformly destroyed the protocols spare blood vessels and duct scaffolds. The healing process is unusually rapid. An additional study has shown that non-thermal IRE can produce focal tissue ablation in the prostate, that the cells are uniformly destroyed in the entire treated region and that the procedure can spare the urethra and the nerves.[15] The safety of the procedure as well as the wide range of possible clinical applications of this molecular mode surgery has been shown through the use of NTIRE for surgical epicardial atrial ablation in a large animal model.[16]

In relation with the topic of the current manuscript our group has recently shown that NTIRE attenuated neointimal formation following angioplasty in a rodent model.[17] We have also demonstrated the long term ablation effect of this modality on VSMC population in rodent carotid artery model.[18]

NTIRE ablation efficacy is dependent on electric pulse parameters (number, length, frequency, magnitude and pulse shape). The electric field effect also depends on electrode design, cell morphology and its orientation, and extra cellular matrix properties.[19]–[21] Therefore, NTIRE effect should be evaluated separately for different tissues. To date, the effect of different NTIRE parameters on VSMC population has not been evaluated. The objective of the current study was to compare the effect of eight different NTIRE protocols on the VSMC population.

## Methods

Thirty three Sprague-Dawley rats weighting 160–280 grams were used in this study. All animals received humane care from a properly trained professional in compliance with both the Principals of Laboratory Animal Care and the Guide for the Care and Use of Laboratory Animals, published by the National Institute of Health (NIH publication No. 85-23, revised 1985).

Animals were anaesthetized with an intramuscular injection of Ketamin and Xylazine (90 mg/Kg and 10 mg/Kg, respectively). The left common carotid artery of each animal was exposed and a custom made electrode clamp with two parallel disk electrodes was applied on the left common carotid artery as previously described.[17] The custom made electrode clamp consisted of two printed circuit boards (1.5 mm thickness) with disk electrodes (diameter = 5 mm) made of copper (70 µm thickness) plated with gold (manufacturing process by Sierra Proto Express, Sunnyvale, CA).

Animals were divided into eight different groups (Table 1). All groups had their left common carotid artery treated with NTIRE and their right common carotid artery used as a control. NTIRE was performed by applying short electric pulses between the electrodes using a high voltage pulse generator intended for electroporation procedures (ECM 830, Harvard Apparatus, Holliston, MA). Current and voltage were recorded by means of special oscilloscope probes (current probe was AP015 and high voltage probe was ADP305, both from LeCroy Corp). Conductance was obtained for each pulse (mean value of the last 10 µs of the pulse) from these two signals. The procedure was repeated in three successive locations along the common carotid artery, treating approximately 1.5 cm of the left common carotid artery. At the end of the procedure the skin incision was sutured closed and the animals were kept alive for a follow-up period of 7 days.

**Table 1:** Study Groups.

Group	Electric Field [V/cm]	# Pulses	Frequency [Hz]	# Animals
**1**	3500	10	10	5
**2**	1750	10	10	4
**3**	875	10	10	4
**4**	437.5	10	10	4
**5**	1750	45	4	4
**6**	875	45	4	4
**7**	1750	90	4	4
**8**	875	90	4	4

The table shows the eight different electroporation parameters used in this study. Groups differ in the magnitude of the applied electric field, the number of the pulses and their frequency. All pulses were square pulses, 100 µs in length. A frequency of 10 Hz was used for the 10-pulse protocols, and was reduced to 4 Hz for 45 or 90 pulse protocols to prevent significant heating.

All pulses were 100 µs in length. The number of pulses, the applied electric field, and the frequency of the pulses differed between the groups as summarized in Table 1.

### Histological Assessment

Animals were euthanized with an overdose of Phenobarbital followed by bilateral chest dissection. Gross inspection of carotid arteries was used to identify arterial wall integrity or intraluminal massive thrombus formation. The arterial tree was perfused with 10% buffered formalin, and the left and right carotid arteries were harvested near the bifurcation of the internal and external carotid arteries. The treated segments were cut into two or three consecutive slices. One section from each slice was used for histological analysis. Each slice was fixed with 10% buffered formalin, embedded in paraffin, and sectioned with a microtome (5-µm-thick). Sections were stained with hematoxylin and eosin, and photographed at ×200 magnification. Then, the following parameters of the Tunica Media were quantitatively evaluated: number of VSMC nuclei in each of the three layers, total area and the average thickness (based on 5 different measurements in each section). VSMC concentration was calculated by dividing the total number of nuclei by the measured area of the Tunica Media. The paired t-test method was used to evaluate the statistical difference between the measured areas of the control versus IRE-treated groups.

In addition, selected sections were stained with elastic Van Gieson (EVG) and Masson trichrome in order to evaluate the extra-cellular elastic and collagen fibers, respectively. Immunostaining with CD31 and CD34 antibodies (Pathology Services Inc., Berkeley, CA) was used to evaluate the endothelial layer.

## Results

All 33 animals survived the procedure. During the follow-up period, there were no cases of infection, bleeding at IRE-treated arteries, thrombosis or animal mortality.

### NTIRE VSMC ablation efficiency

**Figure 1:**
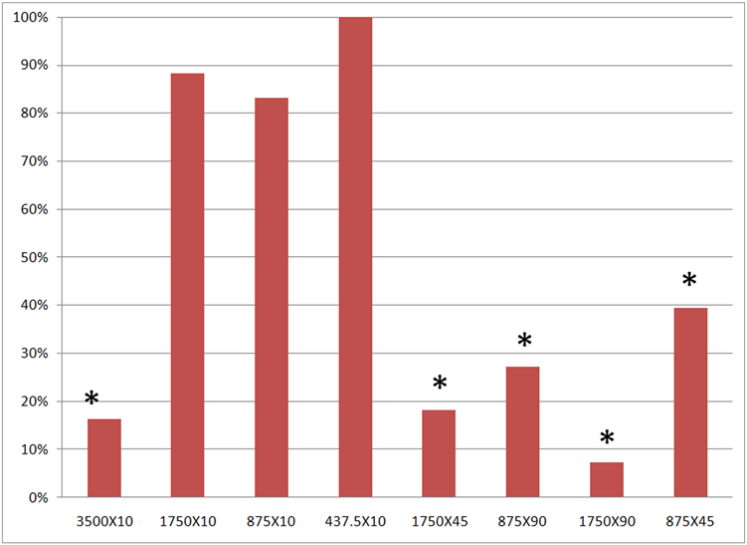
Ablation effect due to different NTIRE protocols. The reduction in five of the groups was statistically significant (P<0.001, bars marked with an asterisk). Ablation effect is shown as the percentage of VSMC cells in the treated artery compared with the right carotid artery of the same animal.

**Figure 2:**
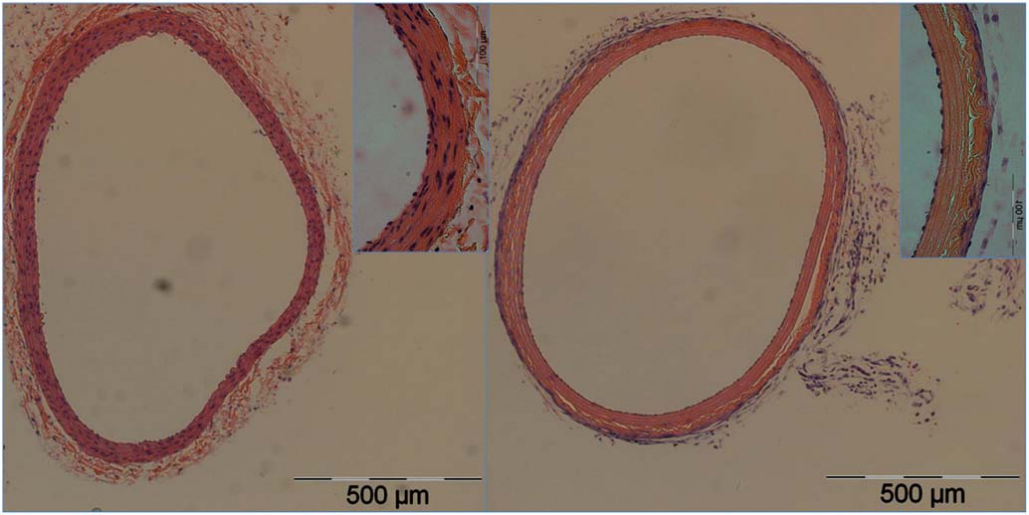
Complete ablation of VSMC population one week following NTIRE. Complete ablation of VSMC population one week following NTIRE with 90 pulses of 1,750 V/cm (right picture) compared with right carotid artery of the same animal that was used as a control (left picture). Note the complete absence of VSMC cells compared with notable repopulation of the endothelial layer with endothelial cells.

Results of all eight groups are summarized in Tables 2–3. Best NTIRE ablation results were achieved in Groups 1 and 7 (Figure 1). Group 1 had 89±16% reduction in the number of VSMC compared with control (24±34 vs. 208±40, P<0.001) and Group 7 had 94±9% reduction in the number of VSMC compared with control (13±21 vs. 213±33, P<0.001). An example of complete ablation of the entire arterial wall is shown in Figure 2.

**Table 2:** Effect of 10 NITRE pulses.

	3500×10 (Group 1)			1750×10 (Group 2)			875×10 (Group 3)			437.5×10 (Group 4)		
	Control	IRE	%	Control	IRE	%	Control	IRE	%	Control	IRE	%
Cell Number	208±40	24±34	11	214±38	167±66	78	196±30	208±58	106	213±38	209±25	98
Concentration	1±0.2	0.2±0.2	16	1±0.2	0.9±0.2	88	1±0.2	0.8±0.3	83	1.3±0.2	1.3±0.2	101
Area	2.2±0.4	1.8±0.3	81	2.1±0.2	1.8±0.3	86	2±0.4	2.8±0.4	136	1.7±0.4	1.6±0.3	98
Thickness	59±8	45±10	75	57±3	54±5	94	58±7	61±9	105	60±6	61±9	102

The table shows data of the four different 10-pulse protocols. All data are shown as average with standard deviation, and include the percentage of IRE values compared with a control. Cell number is the average number of VSMC nuclei identified in the Tunica Media. Concentration is the ratio between the number of cells and the area of the Tunica Media (10^−3^ mm^2^). Area is the total area of the Tunica Media (10^−1^ mm^2^), and the thickness is the thickness of the Tunica Media based on five different measurements in each section in micrometers.

**Table 3:** Effect of 45 or 90 NTIRE pulses.

	1750×45 (Group 5)			875×45 (Group 6)			1750×90 (Group 7)			875×90 (Group 8)		
	Control	IRE	%	Control	IRE	%	Control	IRE	%	Control	IRE	%
**Cell Number**	204±20	30±33	14	230±53	85±66	37	213±33	13±21	6	236±61	49±40	21
**Concentration**	1.5±0.1	0.3±0.3	18	1.6±0.2	0.6±0.4	27	1.5±0.2	0.1±0.2	7	1.6±0.2	0.4±0.3	39
**Area**	1.3±0.1	0.97±0.2	73	1.4±0.2	1.3±0.2	70	1.5±0.3	1.3±0.2	77	1.5±0.2	1.1±0.2	88
**Thickness**	52±3	35±6	67	52±4	47±8	70	51±6	37±4	73	50±4	35±6	90

The Table shows data for the four different protocols with more than 10 pulses. All data are shown in the same manner as in Table 2.

While ten pulses of 3,500 V/cm were efficient, ten pulses of lower electric fields had a minor ablation effect (1,750 V/cm, group 2: 167±66 vs. 214±38, P = 0.05) or no effect at reducing VSMC population (Groups 3 & 4, 875 and 437.5 V/cm respectively). Increasing the number of pulses with an electric field of 1,750 V/cm improved the ablation efficiency (VSMC population reduction of 22±30%, 86±16% and 94±9% with 10, 45 and 90 pulses respectively). A similar trend of increasing efficiency values was also apparent with an electric field of 875 V/cm (63±29% and 79±17% with 45 and 90 pulses, respectively), but efficiency values were not high enough even with 90 pulses (49±40 vs. 236±31, P<0.001).

**Figure 3:**
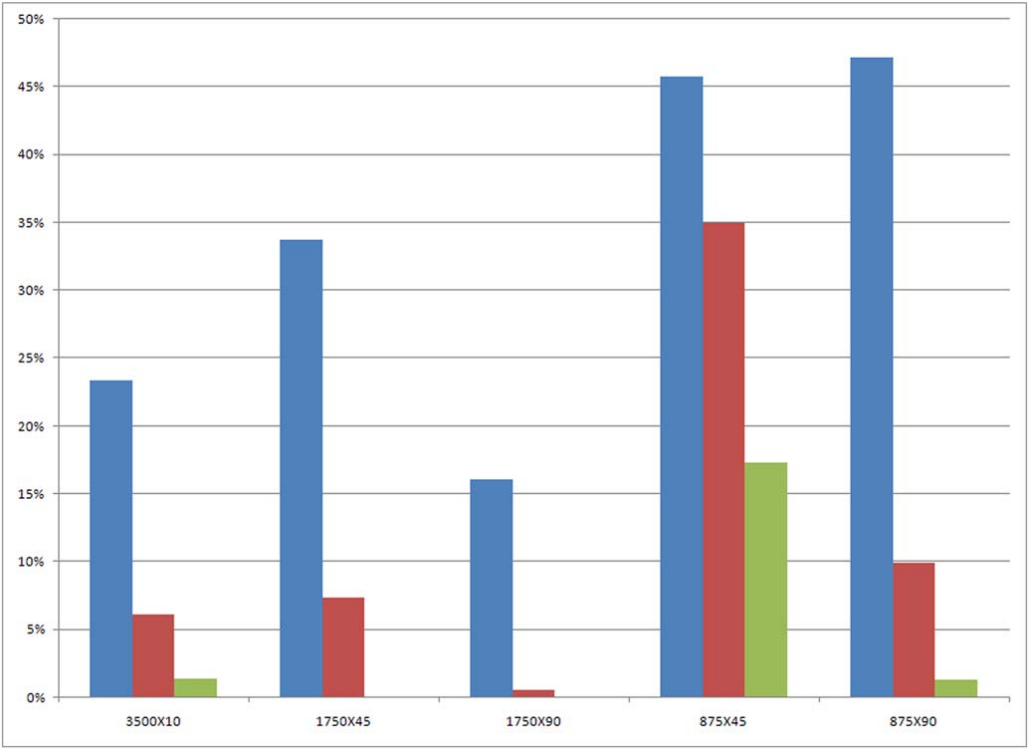
Ablation effect of the sub-layers of the Tunica Media. Inner most, middle and outer sub-layers are in blue, red and green, respectively. Ablation effect is shown as the percentage of VSMC cells in the sub-layer compared with the same sub-layer in the right carotid artery of the same animal. Note the relative sparing of the inner most VSMC cells in all five groups, compared with the complete ablation of VSMC in the outer layers with 1750 V/cm (second and third groups in the figure).

**Figure 4:**
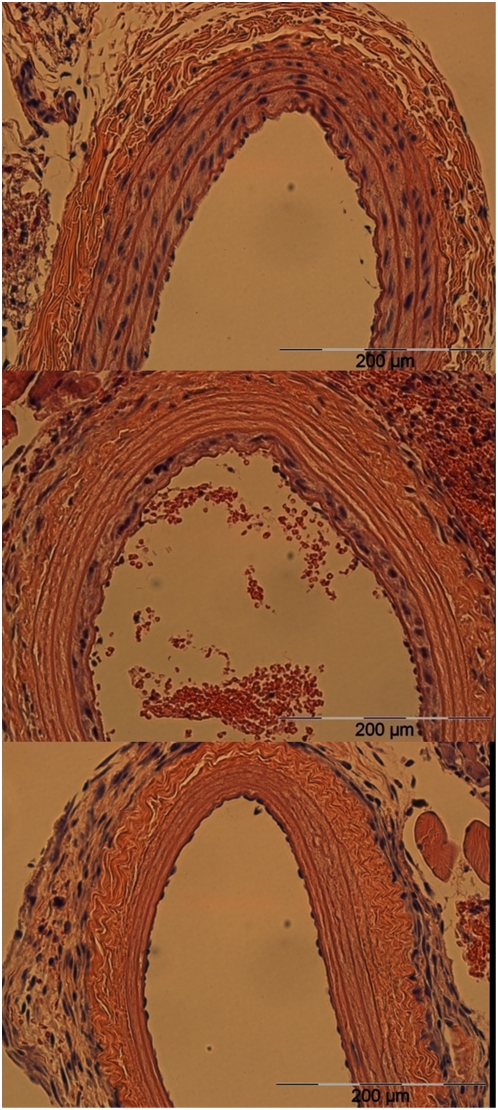
Effect of NTIRE on blood vessels after one week. Higher magnification (×40) of the effect of NTIRE on blood vessels after one week. Top picture shows a control artery, middle picture shows the partial effect due to 45 pulses of 875 V/cm (Group 6), lower picture shows a complete ablation of the arterial VSMC population. In the case of the partial effect - all surviving VSMC are located in the innermost layer of the Tunica Media. Also, note in the lower picture the repopulation of the endothelial layer with endothelial cells, compared with total absence of VSMC.

Sub-analysis of ablation efficiency at the three separate layers of the Tunica Media showed that the best results were achieved in the outer layers of the Tunica Media, and most VSMC that survived NTIRE were located in the inner most layer (Figure 3). For example, in the case of Group 7, no VSMC nuclei could be located in the outer layer in all sections evaluated. All 6% surviving VSMC in this group were located in the inner layer of the Tunica Media (Figure 4).

**Figure 5:**
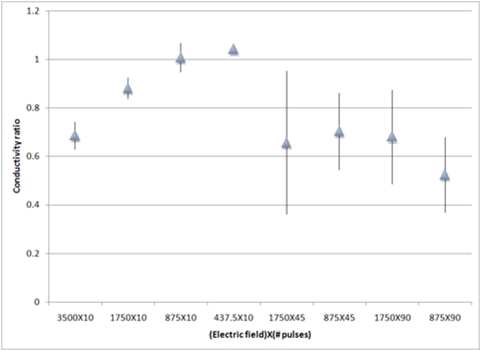
Conductance change during NTIRE application. X-axis shows the eight study groups. Y-axis shows the change as the ratio between the conductance value measured at the last electroporation pulse and the value at the first pulse. Groups 3 and 4 (875×10 and 437.5×10, respectively) show no change in conductivity, which correlates well with the no ablation effect (see figure 2). Group 2 (1,750×10) shows partial reduction in conductivity, correlating well with minor ablation effect.

Electric conductance changed during the application of NTIRE (Figure 5). The conductance measured during the last pulse was lower compared with conductance measured during the first pulse in all groups except Groups 3 & 4 (two groups with no significant NTIRE ablation effect). For the most effective protocols, conductance was reduced by 31±6% and 32±20% for Groups 1 and 7, respectively.

### NTIRE effect on other arterial wall components

Successful NITRE ablation of VSMC induced a reduction in media thickness: 25±17% reduction in group 1 (45±10 vs. 59±8 µm) and 27±7% reduction in Group 7 (37±4 vs. 51±6 µm). No change in media thickness was induced in the two non-successful NTIRE groups (61±9 vs. 58±7 µm in Group 3, 61±9 vs. 60±6 µm in Group 4).

**Figure 6:**
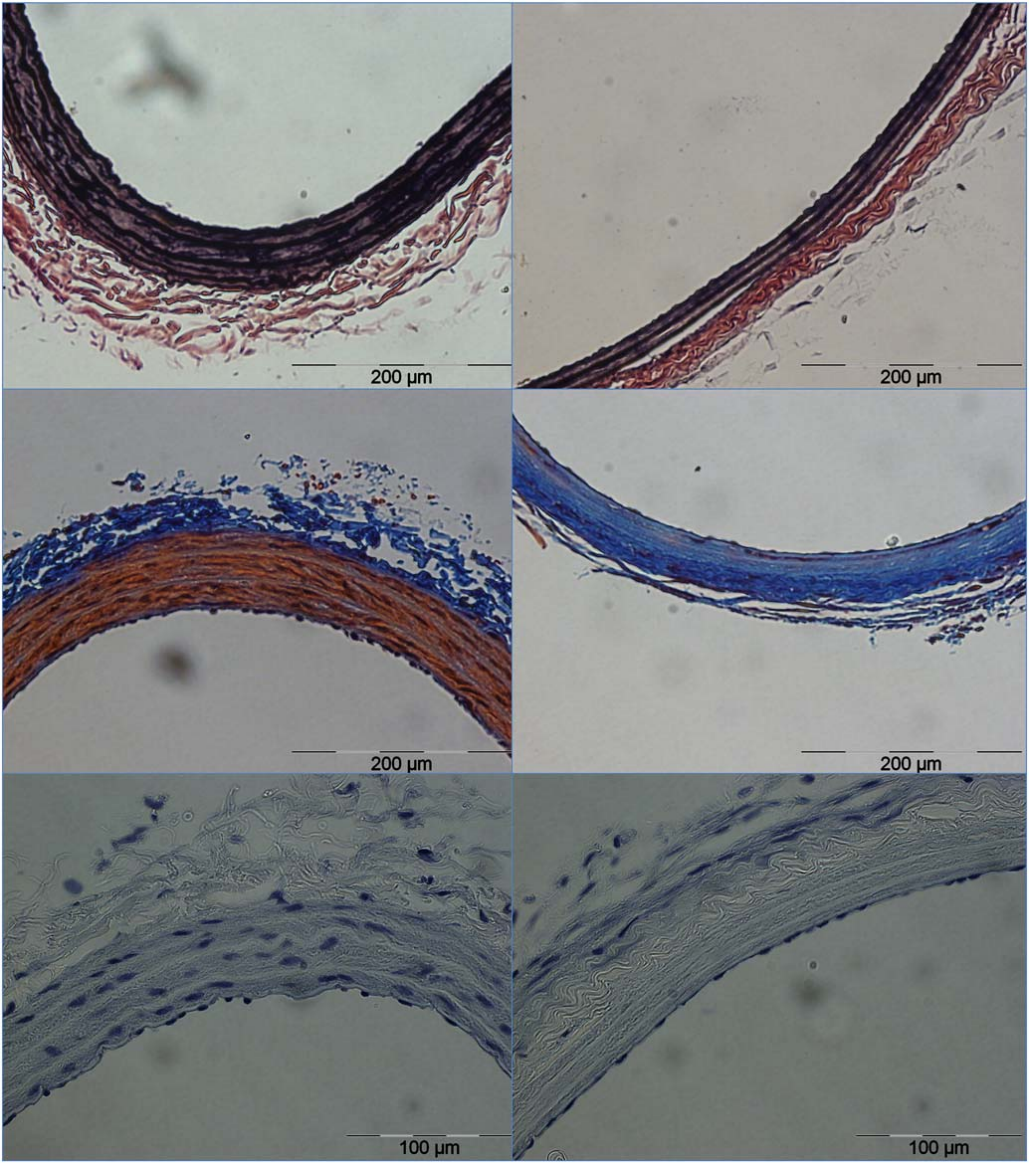
Advanced histology staining. Left column shows control arteries and right column shows IRE-treated arteries. Top row - EVG stain showing undamaged elastic fibers in IRE-treated arteries (elastic Van Gieson, ×40). Middle row - Masson Trichrome stain showing mild fibrosis in the perivascular area with dominance of collagen fibers in the Tunica Media of the IRE-treated Arteries (Masson Trichrome, ×40). Lower row - Negative staining of both arteries with CD34 antibodies at higher magnification (×60). Note the similar morphology and distribution of the endothelial cells.

Endothelial cells of treated arteries were similar in number and morphology to those of non treated control arteries, but were negative to both CD31 and CD34 antibodies (data shown only for CD34 staining, see bottom row in Figure 6). EVG stain demonstrated intact elastic fibers and preserved vessel wall, similar to that of control arteries (middle row, Figure 6). Masson Trichrome stain demonstrated minor fibrosis in perivascular areas, with collagen being the dominant component of the *Tunica Media* following the complete loss of VSMC population (top row, Figure 6).

## Discussion

This is the first large scale, in-vivo survival experiment to evaluate and compare the effect of different NTIRE protocols on VSMC population. The results show that NTIRE can achieve efficient ablation of VSMC within seconds, without damaging extra-cellular components.

Current study results are supported by previous studies by our group. In a preliminary study evaluating NTIRE effect on blood vessels, an 87% reduction in VSMC concentration after 28 days was observed following NTIRE with similar parameters to those of Group 1 (10 pulses, 100 µsec, 10 Hz, 3800 V/cm).[18] Parameters similar to Group 1 have also been shown to significantly reduce neointimal formation following angioplasty in rodent carotid injury model. [17]

Our results are also supported by the work of Al-Sakere et al.[22] In their *in-vivo* study with sarcoma tumor, the best tumor ablation using irreversible electroporation was achieved with the use of 80 electroporation pulses of 100 µs at 0.3 Hz with an electrical field magnitude of 2,500 V/cm. Their most efficient protocol was the one with the largest number of pulses and the highest electric field evaluated, similar to the results presented here. Based on these results, it seems that irreversible electroporation is limited only by the joule heating effect. As long as there is no thermal damage to extra-cellular structures, increase in electric field magnitude and pulse number will be translated to larger ablation volume and better ablation efficiency.

For Groups 1 and 7, where the best ablation efficiency was observed, around 10% of the VSMC population survived the ablation. Further analysis of the results demonstrated 100% efficiency in the outer layers of the Tunica Media, with all surviving cells were located in the inner most layers of the arterial wall (Figures 3–4). The most probable explanation for this phenomenon is the nature of the electric field. We assumed a uniform electric field between the two electrodes, but since the arterial tissue is not homogeneous with respect to its electric properties, the actual electric field in the inner most area of the artery might have been lower than expected. A better design with a more uniform electric field might allow NTIRE to achieve higher ablation efficiencies compared with those reported in this study. Another plausible explanation is the proximity of surviving cells to the oxygenated blood of the carotid artery. The availability of oxygen and nutrient might have a protective effect that reduces the vulnerability of these cells to the stress insult caused by the damage to the cell's membrane.

Our results show that reduction of the electric field magnitude can be compensated by increasing the number of NTIRE pulses. Ten pulses of 3500 V/cm achieved a similar effect to 90 pulses of 1750 V/cm. However, decreasing the electric field to 875 V/cm caused a decrease in NTIRE efficiency even with the use of 90 pulses. This important observation will be critical in future NTIRE device designs, where intervention time could be reduced by increasing trans-electrode electric potential.

A common observation in previous electroporation studies, either reversible or irreversible, is that electrical conductance measured at the pulses increases during the sequence of pulses.[23] The only exception to this seems to be for skeletal muscle under NTIRE.[23] In that particular case, conductance measured at the pulses remains quite constant during the whole sequence and it can be explained as a saturation effect of the electroporation phenomenon. However, the fact that conductance decreases during the sequence is quite surprising as it contradicts what would be expected in a simple electroporation model: electroporation increases cell membrane permeability to ions and therefore its conductance should also increase. We do not have a definitive explanation for the conductance decrease observed here. We consider that a plausible hypothesis is that NTIRE pulses cause a contraction of the arteries by stimulating the vascular smooth muscle cells and that such a contraction results in an increase in the electrical impedance of the arteries.[24]–[26] Another explanation could be based on the fact that electroporation disturbs the osmotic balance of the cells and causes cell swelling which in turn can result in a decrease of the conductance.[23] Nevertheless, we believe that such swelling cannot be manifested as fast as would be required here in order to explain the conductance decrease during the sequence, particularly in Groups 1 and 2 (sequence duration = 1 second).

NTIRE is not the first method to address the challenge of VSMC ablation. Several alternative technologies have been studied, and some have become a common clinical practice for the treatment of post-angioplasty and in-stent restenosis. These technologies include: cryoplasty[27], brachytherapy[28], photodynamic therapy[29], radiofrequency ablation[30], drug-eluting stents[31] and molecular-based therapies[32].

However, delayed re-endothelialization[33], economic impact[34] and late in-stent thrombosis[6], [35] are some of the major concerns with all of the current VSMC ablation modalities. We believe NTIRE should be further investigated as an alternative to current modalities, since it has two major advantages.

First, its non-pharmacological nature can overcome biological phenomena such as cellular adaptation or acquired drug-resistance, thus achieving higher local efficiency. The non pharmacologic nature also guarantees an accurate local effect that depends entirely on electric field distribution and does not induce collateral damage to adjacent structures.

Second, its ultra short duration can decrease intervention time in the clinical setting of primary percutaneous intervention (PCI). It enables one to minimize obstruction of blood flow to viable myocardial tissue during the ablation procedure. Moreover, short intervention duration will enable prompt and full endothelium recovery by immediate recruitment of circulating progenitor endothelial cells. Incomplete neointimal coverage has been demonstrated as a probable cause for late stent thrombosis in patients with drug-eluting stents[6], as well as a reason for brachytherapy failure.[36]

The complete endothelial regeneration observed in this report can be attributed to two properties of NTIRE. First, the ultra short duration of the modality enabled immediate repopulation of the endothelium by either endothelial cells from adjacent non treated areas, or by adherence of progenitor endothelial cells from the circulation. Second, the non-thermal nature of this modality minimized the insult to extra cellular components of the endothelial layer, probably creating a more comfortable environment for cellular regeneration.

Endovascular NTIRE has clinical potential for both the prevention and the treatment of restenosis following angioplasty. Due to its short duration and high efficiency NTIRE can become a preventive treatment immediately before stent deployment. It might also prove to be a valuable tool for the effective treatment of in-stent restenosis following the angioplasty.

### Limitations

We chose to evaluate all animals after a follow-up period of one week. This was based on our previous study, where ablation efficiency was evident by the complete loss of VSMC population as early as one week following NTIRE.[18] However, further studies are needed in order to better understand the long term effect of different NTIRE protocols on vessel wall integrity and strength, arterial modeling and VSMC recovery.

### Conclusion

This study provides scientific proof and justification for further evaluation of irreversible electroporation as a promising non-thermal, non pharmacological, ultra short modality for the treatment of VSMC proliferation and the clinical problem of in-stent restenosis.
